# Exploring the Distinction Between Jinn Possession and Serious Mental Disorders Through the Lens of the Traditional and Faith-Based Healers in Korail Slum

**DOI:** 10.1192/bjo.2024.86

**Published:** 2024-08-01

**Authors:** Tanjir Rashid Soron, Kazi Shammin Azmery, Helal Uddin Ahmed, Jilka Sagar

**Affiliations:** 1 Telepsychiatry Research and Innovation Network Ltd, Dhaka, Bangladesh; 2 NiHealth Ltd, Dhaka, Bangladesh; 3 National Institute of Mental Health (NIMH), Dhaka, Bangladesh; 4 University of Warwick, Warwick, United Kingdom

## Abstract

**Aims:**

The cultural narratives around Jinn Possession are deeply intertwined with the societal understanding of mental health in Bangladesh, often blurring the lines between supernatural beliefs and clinical psychiatric diagnosis. This study aims to delineate the community-based differentiation between Jinn Possession and serious mental disorders such as schizophrenia, bipolar mood disorders and major depressive disorders with psychotic symptoms, as perceived by traditional and faith-based healers in Korail slum. We attempted to unravel the nuanced approaches the healers use to distinguish spiritual afflictions from psychiatric conditions and to explore potential collaborations between traditional healing practices and biomedical mental health services as a part of TRANSFORM Research.

**Methods:**

Adopting an ethnographic and participatory approach, this study engaged in a comprehensive qualitative exploration involving community engagement meetings, 45 key informant interviews, 8 naturalistic interviews with 56 participants, year-long observations of the community and healing practices, 5 co-designing workshops with 46 participants, and 2 pilot training programmes from 2021 to January 2024. We discussed with the traditional and faith-based healers, community health workers, medicine sellers, person with lived experience and their caregivers. The continuous discussion and observation of the community help us to develop a trusted relation and explore the healing practices in the korail slum. Data collected from interviews and workshops were meticulously transcribed and analysed using NVivo software to uncover underlying patterns and distinctions made by traditional and faith-based healers in diagnosing Jinn Possession versus serious mental disorders.

**Results:**

We found a stepwise diagnostic framework utilized by healers, initially categorising conditions based on the symptom's onset and presentation. Sudden and rapid symptoms onset, especially during specific times of the day, was often attributed to Jinn Possession. Specific symptoms such as sudden onset convulsions, disorganised speech and self-laughing further supported this distinction. Moreover, they used traditional diagnostic tests, including the use of holy water and recitation of the Quran, if the patient improves immediately following these interventions was considered as confirmation of Jinn Possession. We observed a few of the healers refer cases perceived as non-spiritual to biomedical facilities when they confirmed it was not the case of Jinn Possession, indicating a potential for collaborative mental health-care models

**Conclusion:**

This cultural understanding offers a unique perspective on community-based mental health care in Bangladesh, emphasising the importance of integrating traditional and biomedical approaches to foster a more inclusive and culturally sensitive mental health-care ecosystem.

